# Preclinical Assessment of Ursolic Acid Loaded into Nanostructured Lipid Carriers in Experimental Visceral Leishmaniasis

**DOI:** 10.3390/pharmaceutics13060908

**Published:** 2021-06-19

**Authors:** Jéssica Adriana Jesus, Ilza Maria Oliveira Sousa, Thays Nicolli Fragoso da Silva, Aurea Favero Ferreira, Márcia Dalastra Laurenti, Leila Antonangelo, Caroline Silvério Faria, Paulo Cardoso da Costa, Domingos de Carvalho Ferreira, Luiz Felipe Domingues Passero

**Affiliations:** 1 Laboratório de Patologia e Doenças Infecciosas (LIM50), Departamento de Patologia, Faculdade de Medicina da Universidade de São Paulo, Av. Dr. Arnaldo, 455-Cerqueira César, 01246-903 São Paulo, Brazil; jessica.adriana@fm.usp.br (J.A.J.); thays.nicolli@gmail.com (T.N.F.d.S.); aurea.favero@gmail.com (A.F.F.); mdlauren@usp.br (M.D.L.); 2 Faculty of Medical Sciences, University of Campinas-UNICAMP, Rua Tessália Vieira de Camargo, 126, Campinas, 13083-871 São Paulo, Brazil; ilzamo.sousa@gmail.com; 3 Laboratório de Patologia Clínica, Departamento de Patologia, Hospital das Clinicas, Faculdade de Medicina da Universidade de São Paulo, Av. Dr. Enéas Carvalho de Aguiar, 155-Cerqueira César, 05403-000 São Paulo, Brazil; l.antonangelo@hc.fm.usp.br (L.A.); carolmbio@gmail.com (C.S.F.); 4 Laboratório de Investigação Médica (LIM03), Hospital das Clínicas, Faculdade de Medicina, Universidade de São Paulo, Av. Dr. Arnaldo, 455-Cerqueira César, 01246-903 São Paulo, Brazil; 5 UCIBIO, REQUIMTE, MEDTECH, Laboratory of Pharmaceutical Technology, Department of Drug Sciences, Faculty of Pharmacy, University of Porto, Rua Jorge de Viterbo Ferreira, 228, 4050-313 Porto, Portugal; pccosta@ff.up.pt (P.C.d.C.); domingos@ff.up.pt (D.d.C.F.); 6 Institute of Biosciences, São Paulo State University (UNESP), Praça Infante Dom Henrique, s/n, São Vicente, 11330-900 São Paulo, Brazil; 7 Institute for Advanced Studies of Ocean, São Paulo State University (UNESP), Rua João Francisco Bensdorp, 1178, São Vicente, 11350-011 São Paulo, Brazil

**Keywords:** nanoparticles, nanostructured lipid carriers, ursolic acid, toxicity, visceral leishmaniasis

## Abstract

Ursolic acid, a triterpene produced by plants, displayed leishmanicidal activity in vitro and in vivo; however, the low solubility of this triterpene limits its efficacy. To increase the activity of ursolic acid (UA), this triterpene was entrapped in nanostructured lipid carriers (UA-NLC), physical-chemical parameters were estimated, the toxicity was assayed in healthy golden hamsters, and the efficacy of UA-NLC was studied in experimental visceral leishmanisis. UA-NLC exhibited a spherical shape with a smooth surface with a size of 266 nm. UA-NLC displayed low polydispersity (PDI = 0.18) and good colloidal stability (−29.26 mV). Hamsters treated with UA-NLC did not present morphological changes in visceral organs, and the levels of AST, ALT, urea and creatinine were normal. Animals infected with *Leishmania (Leishmania) infantum* and treated with UA-NLC showed lower parasitism than the infected controls, animals treated with UA or Amphotericin B (AmB). The therapeutic activity of UA-NLC was associated with the increase in a protective immune response, and it was associated with a high degree of spleen and liver preservation, and the normalization of hepatic and renal functions. These data indicate that the use of lipid nanoparticles as UA carriers can be an interesting strategy for the treatment of leishmaniasis.

## 1. Introduction

Leishmaniasis is a neglected tropical disease, caused by a parasite of the genus *Leishmania* that is transmitted to mammalian hosts during a phlebotomine vector blood meal. Leishmaniasis has been considered a serious public health problem, given the wide geographical distribution, the number of pathogenic species to humans and other vertebrates, diversity of clinical forms, and the scarcity of drugs available for therapy [1,2].

The effectiveness of leishmaniasis treatment depends on several factors, such as drug choice, host immune response, parasite strain, treatment regimen, as well as patient compliance [3]. The treatment of leishmaniasis is not specific to each species nor to the clinical forms and it is performed mainly with pentavalent antimonials. Miltefosine, pentamidine, paromomycin, and amphotericin B are considered second-choice drugs and complete the arsenal to treat all clinical forms of leishmaniasis [4]; however, these drugs have limitations due to side effects, high costs, and in some cases reduced efficacy, sometimes caused by resistant parasites [5,6]. Thus, it is essential to characterize new, efficient, affordable, and less toxic drugs or approaches to combat this neglected disease.

Studies have shown that medicinal plants and their purified molecules, present attractive features to develop new antileishmanial agents, such as selectivity, low toxicity to the experimental hosts, and importantly, high leishmanicidal potential [7]. In this regard, it has been demonstrated that the triterpene ursolic acid (UA) is an interesting antiprotozoal agent, since it is active towards *Leishmania* sp., *Trypanosoma cruzi,* and *Toxoplasma gondii* [8,9,10]. In leishmaniasis, UA eliminated the promastigote and amastigote forms of *L. (L.) amazonensis*, *L. (L*.) *braziliensis*, *L. (L.) donovani, L. (L.) guyanensis,* and *L. (L.) infantum* [11,12,13], suggesting a multispectral activity. Furthermore, it displayed therapeutic activity in experimental cutaneous and visceral leishmaniasis [10,14]. Altogether, these studies show that UA is an interesting molecule to design rational methodologies to improve the treatment of leishmaniasis.

Although the activity of this triterpene has been recorded in experimental leishmaniasis, the main drawback of UA is related to the low solubility in common physiological diluents, which may indeed impact its efficacy in the treatment of leishmaniasis. To overcome this disadvantage, UA can be encapsulated into different types of nanocarriers, which would increase the solubility and availability of this molecule in vivo. Additionally, it is well recognized that drug delivery using nanocarriers can overcome physiological barriers, such as the lipidic membrane of cells, and thus a high concentration of drug will be accumulated in the cytosol of cells as well as the phago-lysosomal compartment. This strategy of treatment may improve the efficacy of the therapy and reduce the toxicity, as the drug release happens at specific locations [15,16]. Among the different drug delivery systems, nanostructured lipid carriers (NLCs) are one of the most studied and show attractive attributes meeting the requirements of an ideal carrier system for lipophilic molecules, such as UA triterpene. The low cytotoxicity, high drug payload, and ability to passively target and release bioactive substances at the site of action are some of the advantages that promote NLCs as carriers of interesting drugs for the treatment of leishmaniasis [17].

Considering the scarcity of safe and effective approved drugs for the treatment of leishmaniasis and the activity of UA towards *Leishmania* species, this study aimed to develop a nanocarrier loaded with UA to enhance the effectiveness of this triterpene in experimental visceral leishmaniasis caused by *L. (L.) infantum.*

## 2. Materials and Methods

### 2.1. Materials

Solid lipid cetyl palmitate (CP) was provided by Gattefossé SAS (St Priest, France), the liquid lipid miglyol 812, and polysorbate 80 (Tween^®^ 80) were purchased from Acofarma^®^ (Madrid, Spain). UA (purity ≥ 98%) and AmB (purity ≥ 99%) were purchased from Cayman chemical company (Ann Arbor, MI, USA) and Cristalia Laboratory (São Paulo, Brazil), respectively. For the HPLC assay, acetonitrile was obtained from VWR (Radnor, PA, USA), ultrapure water (type 1, Milli-Q^®^) was obtained from EMD Millipore (Billerica, MA, USA). Cell culture media were bought from Sigma-Aldrich (Darmstadt, Germany).

### 2.2. Preparation Method of Nanostructured Lipid Carriers (NLCs)

NLC and UA-NLC nanocarriers were prepared by high-pressure homogenization technique. CP (solid lipid—2%) with the mygliol-812 (liquid lipid—3%) and an aqueous solution of polysorbate 80 were heated to 70 °C, separately. Then, UA (0.1%) was dissolved in the lipid phase and the aqueous phase was added. The emulsion was submitted to a homogenization process in Ultra-Turrax T25, with S25N—18G dispersing element (IKA^®^-Labortechnik, Staufen, Germany) at a stirring speed of 10,400 rpm for 5 min. For the NLC formation, the prepared emulsion was quickly transferred to the high-pressure hot homogenizing equipment (High-Pressure Homogenizer SPCH-10, Stansted Fluid Power), being homogenized during five cycles at 600 bar, and then cooled to 25 °C in an ice bath. The samples were stored in glass bottles at 4 °C. Empty NLC was prepared similarly, without adding UA.

In all experiments, UA was quantified using the UltiMate 3000 HPLC apparatus (Dionex Corporation, Sunnyvale, USA), with a UV-VIS detector, and automatic injector. A reverse-phase C18 column (BDS-Hypesil-C18^®^, Thermo Scientific, Waltham, MA, USA) was used with acetonitrile: water (88:12, *v*:*v*) mixture as the mobile phase, with a flow rate of 1.0 mL·min^−1^; UA was detected at 210 nm wavelength [18].

### 2.3. Physical-Chemical Characterization of Nanoparticles

#### 2.3.1. Determination of Encapsulation Efficiency

The freshly prepared formulation was diluted in milli-Q water (1:5), filtered through a 5 µm nitrocellulose membrane filter (Millipore, Ireland), and diluted 1:10 in ethanol to extract UA from NLC. The mixture was centrifuged at 4620× *g* at 25 °C for 15 min (Thermo Scientific Heraeus Multifuge X1R Refrigerated Benchtop Centrifuge, Indianapolis, IN, USA), the supernatant collected, and filtered through a 0.45 µm PTFE syringe filter (Millipore, Ireland). The supernatant was diluted (1:6) in eluent solution and applied to the HPLC column and the amount of *UA* released quantified. The encapsulation efficiency (*EE*) was calculated according to the following equation:

$$EE\;\%=\frac{Amount\;of\;UA\;in\;the\;filtered\;formulation}{Total\;amount\;of\;UA}\times 100$$

#### 2.3.2. Determination of Size, Polydispersity, and Zeta Potential

Particle size and distribution (polydispersity index) were analyzed by Dynamic Light Scattering (DLS, ZetaPALS, Brookhaven Instruments, Holtsville, NY, USA). The zeta potential (ZP) of NLC dispersions was measured by Electrophoretic Light Scattering in a zeta potential analyzer (ZetaPALS, Brookhaven Instruments, Holtsville, NY, USA). Samples were diluted (1:200) in milli-Q water, yielding a suitable scattering intensity. The samples were analyzed at room temperature, with a fixed light incidence angle of 90°; the mean hydrodynamic diameter (*Z*-average), PDI, and ZP were obtained by calculating the mean value of six measurements, performed in three samples.

#### 2.3.3. Morphological Analysis of UA-NLC

The morphology of the NLC was analyzed by transmission electron microscopy JEOL JEM 1400 (Tokyo, Japan). An aliquot of nanoparticles (10 μL) was placed on nickel gratings with Formvar mesh/carbon film (Electron Microscopy Sciences, Hatfield, PA, USA). Samples were contrasted with 1% uranyl acetate solution. Samples were analyzed under a microscope at a voltage of 120 kV. Images were recorded using a CCD digital camera Orious 1100 W Tokyo, Japan.

### 2.4. Animal and Ethical Considerations

Golden hamsters (*Mesocricetus auratus*), 8 weeks old, were obtained from the Anilab (Paulinia, São Paulo, Brazil). This study was performed in accordance with the recommendations of the guide for Care and Use of Laboratory Animals of the Brazilian National Council of Animal Experimentation. The Ethics Committee of Animal Experiments of the Institutional Committee of Animal Care and Use at the Faculdade de Medicina da Universidade de São Paulo (FMUSP) approved the following protocol 056/16, from 22 July 2016. Hamsters were housed in the Animal Experimental Instituto de Medicina Tropical da Universidade de São Paulo (IMTUSP), according to the standards of the Committee of Animal Welfare.

### 2.5. Histological and Biochemical Changes of Healthy Hamsters Treated with UA-NLC

Healthy golden hamsters were divided into 7 groups containing 5 animals/group. The experimental groups were arranged as follows: Groups 1 and 2 were treated with NLC containing 1.25 and 5.0 mg/kg UA, respectively. Groups 3 and 4 were treated with 1.25 and 5.0 mg/kg of UA, respectively; Group 5 was treated with empty NLC (122.5 mg—the equivalent amount of NLC present in Group 2); Group 6 was treated with 5.0 mg/kg of AmB [19] and Group 7 consisted of healthy animals that received only the vehicle solution (PBS control plus 1% DMSO). Animals were treated by the intraperitoneal route, once a day, for 10 consecutive days. One week after the last injection, animals were anesthetized with intraperitoneal sodium thiopental (1 mg/200 µL) and euthanized after total blood collection. The blood was collected in 2 mL tubes without anticoagulants and centrifuged at 3000 rpm, 10 min, 4 °C. The serum was collected and placed in eppendorffs; aliquots of 5 μL were used to analyze the following biochemical parameters: serum alanine transaminase (ALT), aspartate aminotransferase (AST), creatinine, and urea by colorimetric method on COBAS C111 equipment (ROCHE, Indianápolis, USA), as recommended previously by Spada and collaborators [20]. Fragments of the spleen, liver, kidney, lung, and heart were collected, fixed in buffered formalin 10%, processed using usual histological techniques, and 3 μm thick sections were stained with Hematoxylin and Eosin (HE).

### 2.6. Analysis of the Therapeutic Potential of UA-NLC

*L. (L.) infantum* (MHOM/BR/72/46) was provided by Prof. Dr. Fernando Tobias Silveira, from the cryobank of the Leishmaniasis Laboratory Prof. Dr. Ralph Laison, Department of Parasitology, Instituto Evandro Chagas (Para State, Brazil). Parasites were identified using monoclonal antibodies and isoenzyme electrophoretic profiles. *L. (L.) infantum* parasites were maintained in Schneider’s Medium (Sigma Aldrich, Darmstadt, Germany), supplemented with 10% heat-inactivated fetal bovine serum and 50,000 IU/mL penicillin, 50 μg/mL streptomycin (S10). Stationary phase promastigotes were used throughout the entire study.

Golden hamsters (8 weeks old) were infected intraperitoneally with 2 × 10^7^ *L. (L.) infantum* promastigote forms. The noninfected control group was injected with PBS alone. Infected hamsters were divided into 8 groups, with 5 animals each. After 60 days of infection, the treatment was initiated with UA, UA-NLC, AmB or empty NLC. The experimental groups were arranged as follows: Groups 1 and 2 were treated with UA-NLC, containing 1.25 and 5.0 mg/kg of UA, respectively; Groups 3 and 4 were treated with 1.25 and 5.0 mg/kg of UA, respectively; Group 5 was treated with NLC (122.5 mg—the equivalent amount given to animals from group 2); Group 6 was treated with 5.0 mg/kg of AmB [19]; Group 7 was injected with vehicle solution (PBS plus 1% DMSO—Infected control group) and Group 8 consisted of the non-infected control group and received only vehicle solution. Animals were treated by the intraperitoneal route, once a day, for 10 consecutive days. One week after the last injection, the animals were euthanized and the serum was collected to quantify *Leishmania*-specific IgG and IgG2a by Enzyme-Linked Immunosorbent Assay (ELISA) (Southern Biotech, Birmingham, AL, USA); AST, ALT, creatinine, and urea were quantified by a colorimetric method on COBAS C111 equipment (ROCHE, Indianápolis, IN, USA), as detailed in the Section 2.5. Fragments of the spleen and liver were collected to determine splenic and hepatic parasitism as well as histological changes.

#### 2.6.1. Determination of Parasite Load

The splenic and hepatic parasitism was quantified by limiting dilution assay [10]. Briefly, the spleen and liver were collected, weighted and aseptically homogenized in S10. Spleen and liver suspensions were subjected to 12 serial dilutions in sterile 96-well plates with four replicate wells. The number of parasites was determined based on the highest dilution that promastigote forms could grow after ten days of cultivation at 25 °C. Additionally, parasitism in both organs was evidenced by the immunohistochemistry technique [21].

#### 2.6.2. Analysis of Cellular and Humoral Immune Responses

RNA from hamster spleen fragments (~10 mg) was purified using the commercial RNeasy Mini Kit (Qiagen, Hilden, Germany). Isolated RNA was used to synthesize cDNA with the SuperScript^®^VILO™ cDNA Synthesis Kit (Life Technologies Carlsbad, CA, USA). Amplification conditions consisted of an initial denaturation phase at 95 °C for 10 min, followed by 40 amplification cycles consisting of 95 °C for 15 s (s); 61 °C for the 90 s, and 72 °C for 30 s. All reactions were performed in a Mastercycler Nexus GSX1 (Eppendorff, Framingham, MA, USA). Before quantification, the efficiency of each reaction was verified using cDNA from the spleen of healthy animals; that was always above 95%. β-actin (endogenous control) was used to normalize the expression level of the genes. qPCR reactions were performed using the GoTaq^®^ 1-Step RT-qPCR System (Promega Corporation, Madison, WI, USA) and 75 nM of primers. The primer sequences were as follows (5′ to 3′): IFN-γ forward: GACAACCAGGCCATCC and reverse: CAAAACAGCACCGACT; interleukin -10 (IL-10) forward: TGGACAACATACTACTCACTG and reverse: GATGTCAAATTCATTCATGGC; enzyme inducible nitric oxide synthase (iNOS) forward: CGACGGCACCATCAGAGG and reverse: AGGATCAGAGGCAGCACATC; β-actin forward: TCCTGTGGCATCCACGAAACTACA and reverse: ACAGCACTGTGTTGGCATAGAGGT [22,23,24]. Quantification results were expressed in fold changes of 2^−ΔΔCt^ over the infected control group [25]. PCR products were electrophoresed on 2% agarose to confirm the amplification of products. For each reaction, one single product of predicted size [22] was always obtained.

Soluble antigen of *L. (L.) infantum* was used to analyze the humoral immune response by ELISA. Briefly, promastigote forms in stationary phase of growth were collected by centrifugation at 3000 rpm, 10 min, 4 °C, and the pellet was washed three times with PBS. The pellet was resuspended in 100 μL of PBS and immediately frozen in liquid nitrogen. Following this step, the pellet was thawed at room temperature. This cycle of freeze and thaw was repeated three times, allowing parasite lysis. The lysate was centrifuged at 10,000 rpm, 30min, 4 °C; the supernatant was collected and the concentration of protein was determined using the Bradford method (Biorad, Hercules, CA, USA). Ninety-six-well high-binding ELISA plates (Costar, USA) were coated with the soluble antigen of the promastigote forms of *L. (L.) infantum* (1.0 μg of protein/well) in the carbonate-bicarbonate buffer, pH 9.6 (100 mM NaHCO_3_; 6 mM Na_2_CO_3_), for 18 h at 4 °C. After this period, the wells were washed three times with PBS plus 0.05% Tween 20 (PBST), and nonspecific bindings were blocked with 10% skimmed milk diluted in PBS for 120 min at 37 °C. The wells were washed three times with PBST, and 100μL of animal serum (diluted 1: 1000 in PBST) were added to each well and the plate was incubated for 60 min at 37 °C. After this period, the wells were washed three times with PBST, and the secondary antibodies goat anti-hamster IgG (1:10,000) or IgG2 (1:16,000), both conjugated with horseradish peroxidase—HPR—(Southernbiotech, Birmingham, AL, USA) were added to the wells for 60 min at 37 °C. After this step, the wells were washed five times with PBST, and the substrate 3.3′, 5.5′ tetramethylbenzidine—TMB—(B&D, USA) was added to the wells for 15 min. The reaction was blocked by the addition of 50 μL/well of sulfuric acid (2N) and the absorbances were read in an ELISA reader at 450 nm wavelength. Serum from animals chronically infected with *L. (L.) infantum* and healthy animals were used as positive and negative controls of the reactions, respectively.

### 2.7. Statistical Analyses

Statistical analyses were performed using the GraphPad Prism 5.0 software (San Diego, CA, USA) and the nonparametric test Kruskal–Wallis, followed by Dunn’s multiple comparison test, was used. Differences between two groups were analyzed by an unpaired *t*-test. Differences were considered statistically significant at the 5% level (*p* < 0.05). Values were expressed as mean ± standard deviation from a minimum of three independent experiments.

## 3. Results

### 3.1. Physical Characterization of NLC

The size of nanoparticles (PS), index of polydispersity (PDI), zeta potential (ZP) and efficacy of encapsulation (EE) are shown in Table 1. In general, the mean size of UA-NLC and NLC was below 267 nm. The PDI of UA-NLC was 0.18 while NLC was 0.16 and the value of ZP for UA-NLC was −29.26 mV and NLC was −26.12 mV; the EE of UA was 59.71%.

**Figure 1:**
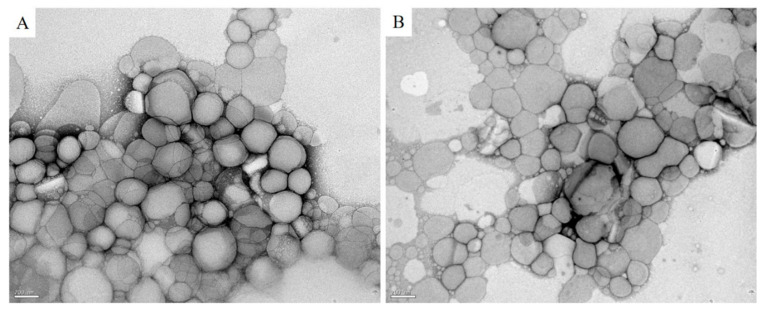
TEM images of the Nanostructured Lipid Carriers (NLC) (**A**) and UA-NLC (**B**). Magnification of 50,000×.

The morphology of the lipid nanoparticles, observed by TEM, revealed that both NLC (Figure 1A) and UA-NLC (Figure 1B) were spherical and uniform in shape with smooth surfaces, corroborating the results shown in Table 1. Additionally, it was possible to observe that, by loading UA into NLC, the morphology of the nanocarrier was not altered in comparison to NLC (Figure 1A,B).

### 3.2. Biochemical and Histological Changes of Healthy Hamsters Treated with UA-NLC

**Figure 2:**
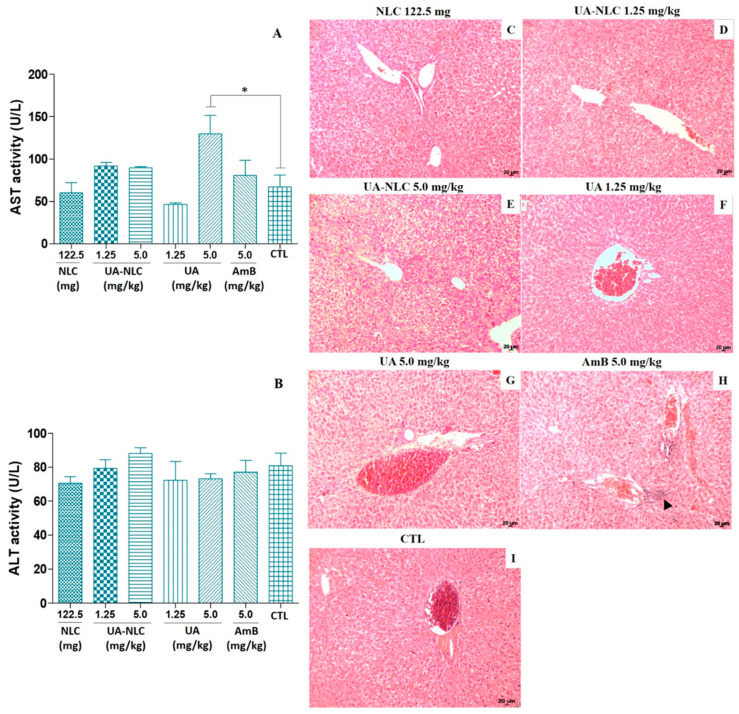
Biochemical and histological changes were analyzed in golden hamsters treated with UA or UA-NLC. Seric levels of AST (**A**) and ALT (**B**); histological sections of the liver from *Mesocricetus auratus* treated with NLC (**C**) or UA-NLC at 1.25 or 5.0 mg/kg (**D**,**E**, respectively) were monitored; UA at 1.25 and 5.0 mg/kg (**F**,**G**, respectively); 5.0 mg/kg of AmB (**H**), and control animals untreated—CTL—(**I**). Hematoxylin-Eosin. 100× magnification. * *p* < 0.05 indicates statistical significance.

Golden hamsters treated with UA-NLC did not show significant changes in the levels of AST (Figure 2A) and ALT (Figure 2B) in comparison with the control group. ALT levels were not altered in animals treated with UA; however, a significant elevation in the levels of AST were observed in animals treated with 5.0 mg/kg of UA (*p* < 0.05), as observed in Figure 2A. In the liver, no histological changes were observed in animals treated with NLC, UA, or UA-NLC (Figure 2C–G). However, inflammatory nodules were observed in the portal areas of the liver from animals treated with AmB (arrowhead in Figure 2H).

**Figure 3:**
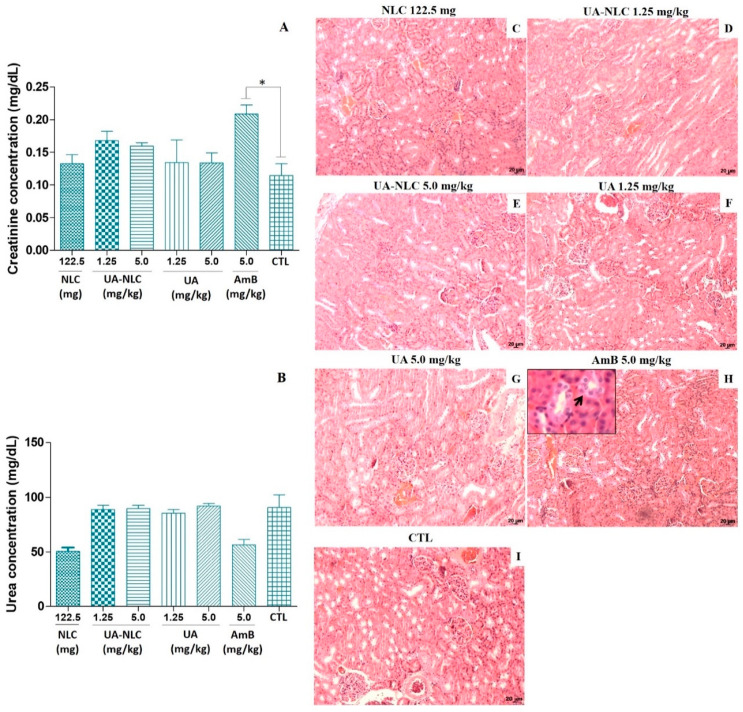
Biochemical and histological changes were analyzed in golden hamsters treated with UA or UA-NLC. Levels of serum creatinine (**A**) and urea (**B**) were estimated, and histological sections of the kidney from healthy *Mesocricetus auratus* treated with NLC (**C**) or UA-NLC at 1.25 or 5.0 mg/kg (**D**,**E**, respectively); UA at 1.25 and 5.0 mg/kg (**F**,**G**, respectively); 5.0 mg/kg of AmB (**H**), and control animals untreated—CTL—(**I**) were monitored. Hematoxylin-Eosin. 100× magnification. * *p* < 0.05 indicates statistical significance.

Experimental animals treated with 1.25 and 5.0 mg/kg of UA or UA-NLCs did not alter the levels of creatinine (Figure 3A) and urea (Figure 3B) in comparison with the control. In contrast, a significant increase in the level of creatinine was detected in animals treated with 5.0 mg/kg of AmB (*p* < 0.05) compared with the control group (Figure 3A). Levels of urea were similar in all experimental groups (Figure 3B). The treatment of animals with UA-NLC or UA did not alter the morphology of the cortical and medullary regions of the kidney (Figure 3D–G). However, in animals treated with AmB, a vacuolization of epithelial cells of the proximal and distal tubules of the medullary region of the kidney was observed (black arrow in Figure 3H).

Hamsters treated with 1.25 or 5.0 mg/kg of UA-NLC, 1.25 or 5.0 mg/kg of UA; 5.0 mg/kg of AmB or NLC did not present significant histological changes in the spleen, lung, and heart (data not shown).

### 3.3. Analysis of the Therapeutic Potential of UA-NLC

**Figure 4:**
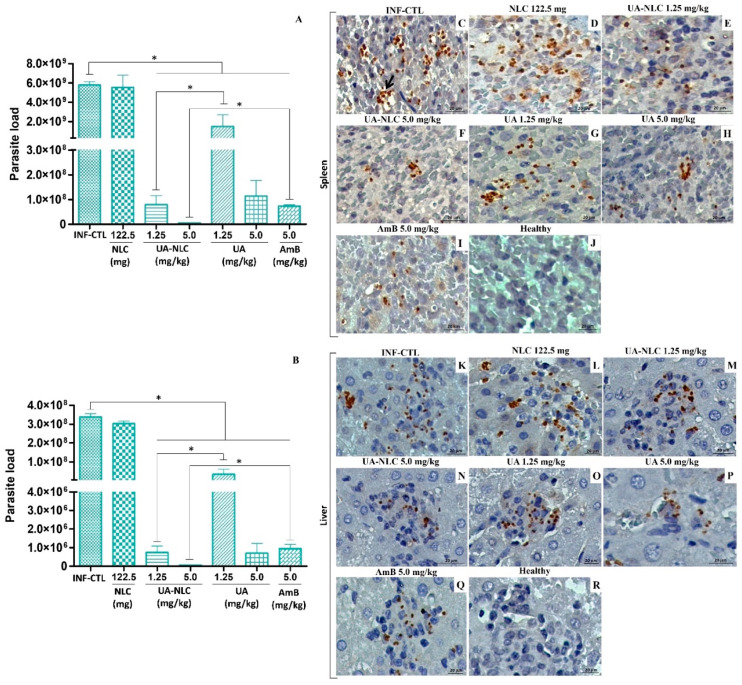
Golden hamsters infected with *L. (L.) infantum* were treated with UA-NLC or UA and the tissue parasitism was analyzed by limiting dilution assay. Parasitic load on the spleen (**A**) and liver (**B**) of animals infected with *L. (L.) infantum* and treated with UA-NLC or UA at concentrations 1.25 and 5.0 mg/kg; AmB was given at 5.0 mg/kg. Photomicrographs of histological sections stained by immunohistochemistry show amastigote forms (stained in dark brown) in the spleen (**C**–**J**) and liver (**K**–**R**) of the infected control group (**C**,**K**), as well as empty NLC (**D**,**L**), UA-NLC (**E**,**F**,**M**,**N**), UA (**G**,**H**,**O**,**P**) or treated hamsters with AmB (**I**,**Q**) (magnification 400; scale bar: 20 μm). * *p* < 0.05 indicates statistical significance.

In the spleen (Figure 4A) and liver (Figure 4B) of animals infected with *L. (L.) infantum* and treated with 1.25 or 5.0 mg/kg of UA-NLC or UA a significant reduction in the number of parasites in comparison with the infected controls was observed (*p* < 0.05). Furthermore, it was observed that UA-NLC (1.25 mg/kg) showed superior therapeutic activity than UA (1.25 mg/kg) at eliminating splenic and hepatic amastigotes (*p* < 0.05). AmB also reduced splenic and hepatic parasites in comparison with the infected control (*p* < 0.05); however, animals treated with 5.0 mg/kg of UA-NLC displayed superior antileishmanial properties than AmB in both organs (*p* < 0.05).

Histological sections of the spleen and liver were immunolabelled to observe amastigote forms (Figure 4C–I and Figure 4K–Q, respectively). In the spleen (Figure 4C,D) and liver (Figure 4K,L) of infected controls, an elevated number of amastigote forms were immunolabeled. In comparison with the controls, a low number of splenic and hepatic amastigote forms were observed in animals treated with 1.25 and 5.0 mg/kg UA-NLC, UA (Figure 4E–H,M–P), or AmB (Figure 4I,Q).

### 3.4. Histopathological Changes in the Spleen and Liver of Animals Treated with UA-NLC or UA

**Figure 5:**
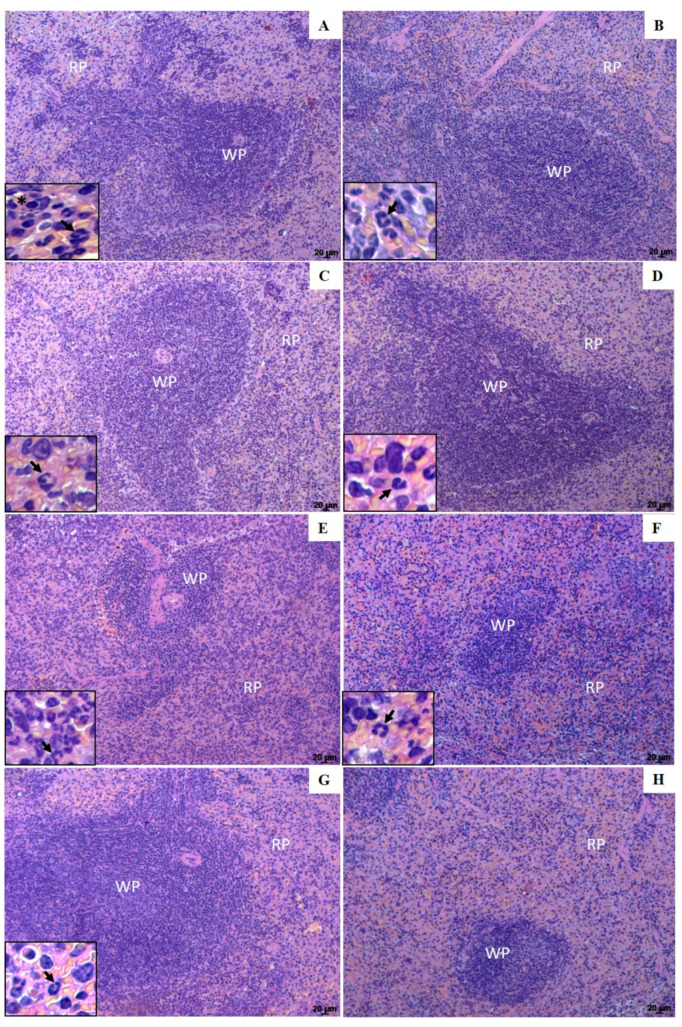
Photomicrographs of histological sections of the white pulp (WP) and red pulp (RP) areas of the spleen from golden hamsters. Infected control (**A**); Infected and treated with empty NLC (**B**); Infected and treated with 1.25 and 5.0 mg/kg UA loaded in NLC (**C**,**D**, respectively), Infected and treated with 1.25 and 5.0 mg/kg of UA (**E**,**F**, respectively) or AmB (**G**). Spleen histological section from healthy animals is shown in image H. Insets show in detail amastigotes forms (*) and polymorphonuclear cells (arrows) of the spleen histological sections. Magnification of 100×; scale bars: 20 μm (**A**–**H**).

Histological sections from the spleen of infected control groups (infected nontreated or treated with empty NLC), shown in Figure 5A,B, respectively, displayed the replacement of lymphoid follicles by infected macrophages (inset Figure 5A,B). Furthermore, polymorphonuclear cells were observed in both control groups, indicating high disease severity. Comparatively, histological sections of the spleen of animals treated with 1.25 and 5.0 mg/kg of UA-NLC showed a low number of parasites and polymorphonuclear cells compared to the infected controls (inset Figure 5C,D), and preservation of the white pulp, suggesting a better host response after treatment (Figure 5C,D, respectively). Animals treated with 1.25 and 5.0 mg/kg of UA (Figure 5E,F, respectively) or AmB (Figure 5G) also showed preservation of the white pulp, expansion of the red pulp marked by the presence of a few parasitized macrophages, lymphocytes and moderate presence of neutrophils (details in the inset of the respective figures). The histological sections of the spleen of healthy animals, on the other hand, had a normal histological aspect, with well-preserved white and red pulp, as shown in Figure 5H.

**Figure 6:**
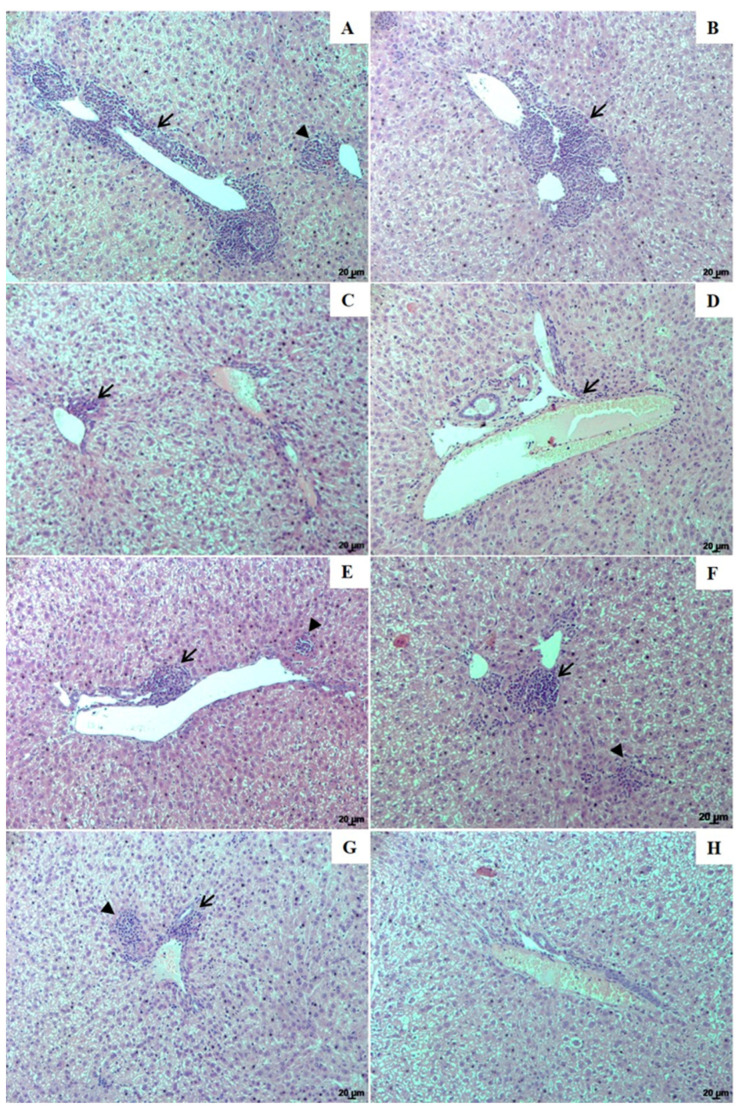
Histological changes of the liver from golden hamsters infected with *L. (L.) infantum*. Liver histological sections from (**A**)—infected control; (**B**)—infected and treated with empty NLC; animals treated with 1.25 and 5.0 mg/kg UA loaded in NLC (**C**,**D**, respectively), animals treated with 1.25 and 5.0 mg/kg free UA (**E**,**F**, respectively) or AmB (**G**). Liver histological sections from healthy animals are shown in image H. Inflammation foci (arrows) and granulomas (arrowhead). Magnification of 100×; scale bars: 20 μm (**A**–**H**).

In the animals from the infected control group, NLC, and those treated with 1.25 and 5.0 mg/kg of UA-NLC or UA, the main histopathological finding in the liver was related to periportal inflammation with the presence of parasitized macrophages, and granulomas in the portal space and parenchyma (Figure 6A–H). Additionally, all infected animals showed hyperplasia and hypertrophy of Kupffer cells, which were sometimes parasitized; however, periportal inflammation, parasitism, and granuloma formation in the parenchyma was less frequent in animals treated with UA-NLC (Figure 6C,D), followed by UA (Figure 6E,F) and AmB (Figure 6G) than infected controls (Figure 6A,B). Healthy animals displayed normal hepatic morphology (Figure 6H).

### 3.5. Analysis of Cellular and Humoral Immune Responses

**Figure 7:**
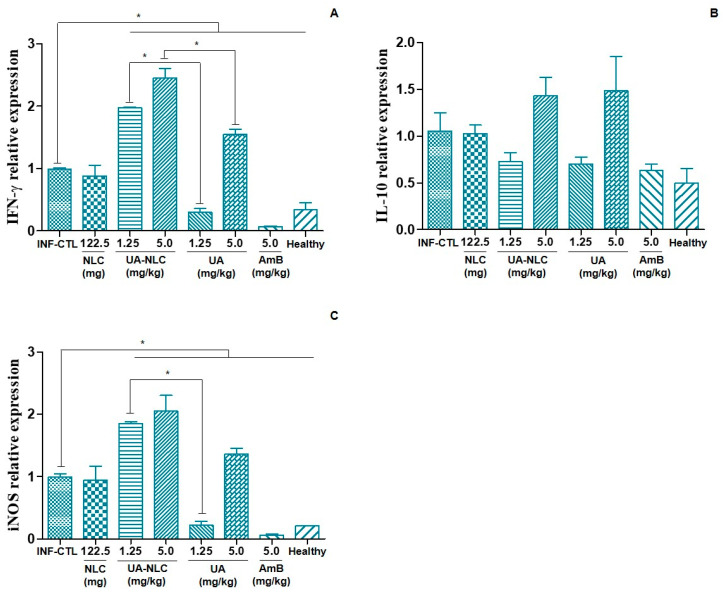
Relative mRNA expression of IFN-γ (**A**), IL-10 (**B**) and iNOS (**C**) in the spleen of control and treated hamsters infected with *L. (L.) infantum*. * *p* < 0.05 indicates statistical significance.

Animals treated with UA-NLC (1.25 or 5.0 mg/kg) or UA (5.0 mg/kg) expressed higher levels of IFN-γ (Figure 7A) than the infected control group (*p* < 0.05). Additionally, it was found that the groups treated with UA-NLC (1.25 or 5.0 mg/kg) expressed higher levels of IFN-γ than animals treated with UA at the same doses (Figure 7A). The expression of IL-10 in the spleen was similar among all analyzed groups (Figure 7B). A significant expression of iNOS gene in the spleen of animals treated with UA-NLC (1.25 or 5.0 mg/kg) or UA (5.0 mg/kg) was verified in comparison to the infected control group (Figure 7C). A significant increase in iNOS expression was observed in the group treated with UA-NLC (1.25 mg/kg) compared to UA at the same dose (Figure 7C).

**Figure 8:**
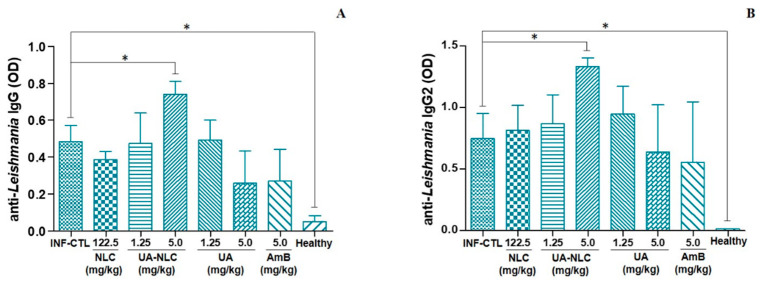
Levels of antileishmanial IgG (**A**) and IgG2 (**B**) in the serum of hamsters infected with *L. (L.) infantum* and subjected to the treatment with 1.25 or 5.0 mg/kg UA loaded in NLC or UA; additionally, animals were treated with 5.0 mg/kg of AmB, as a standard treatment. * *p* < 0.05 indicates statistical significance.

The analysis of the humoral immune response showed that only hamsters treated with 5.0 mg/kg UA-NLC produced a significant amount of antileishmanial IgG (Figure 8A) and IgG2 (Figure 8B) in comparison to the infected control group (*p* < 0.05).

### 3.6. Biochemical Analysis of Hamsters Infected and Treated with UA-NLC

**Figure 9:**
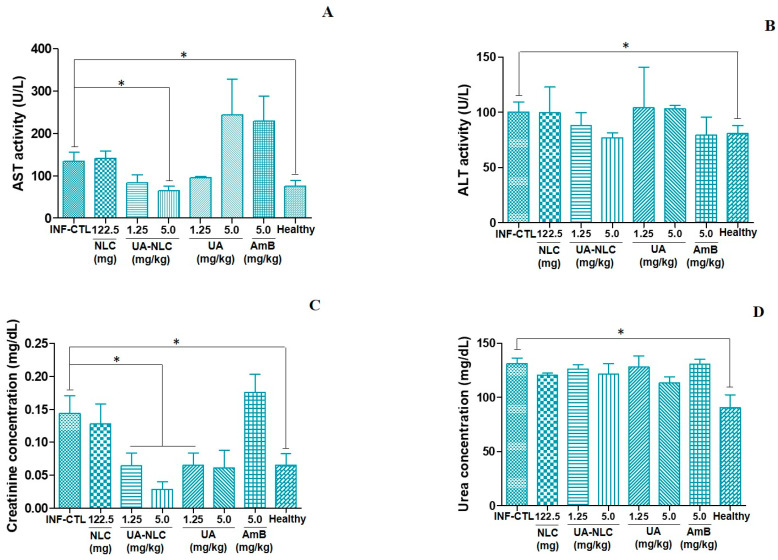
Serum values of AST (**A**), ALT (**B**), creatinine (**C**) and urea (**D**) in infected controls, treated with NLC, UA, UA-NLC, or AmB for 10 consecutive days. * *p* < 0.05 indicates statistical significance.

Animals treated with UA-NLC or UA did not change the levels of AST and ALT in comparison with the infected control (Figure 9A,B); although animals treated with 5.0 mg/kg of UA-NLC displayed a significant reduction in the levels of AST in comparison with the infected control group (*p* < 0.05), and AST level was similar with the healthy animals (Figure 9A). Animals treated with UA-NLC or UA exhibited a significant reduction in the levels of creatinine in comparison to the infected control (*p* < 0.05), and these values were close to the normal levels observed in the healthy group (Figure 9C). Urea levels were similar in all treated animals (Figure 9D), except in the healthy group that showed low levels of urea in comparison with the infected control (*p* < 0.05).

## 4. Discussion

Nanotechnology has been considered an important tool to reduce side effects and increase the effectiveness and selectivity of drugs [26]. In this sense, NLCs have been elected as an interesting drug platform to treat different intracellular infections [27], because they can access the cytoplasm of cells and deliver the content directly into the target. This may account for the high efficacy of treatment performed with nanocarriers than drugs freely administered.

In the present study, high-pressure homogenization was used to formulate UA-NLC that exhibited a monodisperse distribution with a suitable mean size (266 nm) and PDI of 0.18, which indicates a homogenous and narrow size distribution of the nanoformulations. Particles with a size between 100 and 500 nm may be used in human and/or animal therapy, since they remain in circulation longer [28,29], enabling the distribution of nanoparticles through the tissues, including the liver and spleen, which are the main affected organs in visceral leishmaniasis. Furthermore, the steric stabilization on the surface of the nanoparticles led to negative zeta values, −26 and −29 mV for NLC and UA-NLC, respectively, suggesting that the samples remained dispersed and stable, with a reduced tendency to form aggregates due to the electrostatic repulsion [30]. These results are consistent with those obtained by other research groups, which produced NLCs with a similar range of negative zeta values [31,32]. Additionally, transmission electron microscopy showed that the incorporation of UA in NLCs preserved the size and morphology of the nanoparticles. Therefore, the physical properties obtained herein enabled the use of UA-NLC in vivo.

The properties of UA-NLC on the visceral organs and on the metabolism of the liver and kidney of healthy golden hamsters were investigated. In the histopathological study, it was verified that the treatment of hamsters with UA-NLC did not change the morphology of the spleen, liver, kidney, lung or heart. Hepatic and renal functions of animals were also investigated, since these organs are responsible for the metabolization and excretion of drugs [33,34]. UA-NLC did not alter the levels of AST, ALT, urea or creatinine in golden hamsters; however, an increase in the levels of AST was observed in animals treated with 5.0 mg/kg of UA. AST is considered an important marker of structural damage of hepatocytes and is widely used in the assessment of liver damage caused by drugs or hepatotoxins [35]. These data suggest that UA given by the intraperitoneal route at 5.0 mg/kg damaged hepatocytes; however, no structural changes were observed, pointing to an initial damage induced by UA. On the other hand, animals treated with NLC carrying 5.0 mg/kg of UA did not alter the levels of AST, suggesting that UA was delivered to phagocytic cells, avoiding hepatocyte damage [33]. Previous studies showed that hamsters treated for sixteen days with 1.0 and 2.0 mg/kg of UA by the intraperitoneal route did not present biochemical or histopathological changes in the liver, suggesting that UA is safe at low doses [10]. However, UA exhibited mild toxicity at higher doses, that may be abolished after encapsulation.

In experimental models of liver damage, it was observed that UA presented hepatoprotective potential when administered by the oral route [36,37,38]. In the present work, UA given by the intraperitoneal route induced moderate liver toxicity, suggesting that the route of administration and dose may be associated with hepatotoxicity. In a clinical trial, healthy volunteers and patients with solid tumors were treated with a single dose of UA entrapped in liposomes by the intravenous route; although low and mild doses were safe to volunteers, high doses of the formulation induce several side effects, including the elevation of hepatic enzymes [39]. On the other hand, it has been demonstrated that UA has been safely used as a daily dietary supplement, without toxic events [40,41,42]. It is worth remembering that UA is naturally synthesized by different fruits and vegetables present in the human diet [43]; therefore, UA toxicity may be related to the dosage, formulation, duration of treatment and route of administration.

In contrast to UA-NLC, it was observed that AmB caused morphological changes in the medullary area of the kidney along with an increase in creatinine levels in the blood, suggesting that the treated animals developed acute renal failure [44]. A common event in therapy with AmB is nephrotoxicity, which affects 49 to 65% of patients under treatment [45], which may show a reduced glomerular filtration rate, leading to renal dysfunction and failure [46,47]. Similarly, in the hamster model of visceral leishmaniasis, AmB also induced significant morphological and biochemical changes in the kidney [10], suggesting that in animal models, AmB also causes kidney failure. Taken together, these findings demonstrate that UA-NLC were not toxic for golden hamsters, while AmB, although recognized as an important leishmanicidal drug, induced severe side effects in golden hamsters.

In infected golden hamsters, it was verified that animals treated with UA-NLC or UA exhibited a significant reduction in splenic and hepatic parasitism, suggesting that both UA and UA-NLC presented high therapeutic potential. The number of parasites reduced as the UA dose increased, and this effect was maximized when UA was encapsulated. It was observed that treatment with UA at 1.25 mg/kg (cumulative total amount of 1.75 mg) reduced parasitism by 74.20% in the spleen and 90.04% in the liver, while the same dose of UA loaded into NLC decreased splenic and hepatic parasitism by 98.63 and 99.78%, respectively. Animals treated with 5.0 mg/kg of UA (cumulative total amount of 7.0 mg) had a reduction in parasitism by 98.03% in the spleen and 99.79% in the liver, while a reduction of 99.92 and 99.98% in splenic and hepatic parasite loads, respectively, was observed in animals treated with 5.0 mg/kg of UA-NLC. These data suggest that NLC improved the efficacy of UA when compared to UA administered freely, reinforcing that nanoparticles can be good carriers of drugs, as it has been observed in different studies [48,49,50,51]. Das and collaborators [52] demonstrated that UA-loaded NLC coated with chitosan oligosaccharides had high anti-leishmania activity in vitro, and when administered orally to BALB/c infected with *L. (L.) donovani*, this formulation was 94 times more active than miltefosine, 5 times more than sodium stibogluconate and 2 times more than paromomycin. According to a study conducted by Kar and collaborators [53], NLC potentiated the leishmanicidal effect of the sesquiterpene cedrol on amastigotes forms of *L. (L.) donovani* resistant or not to stibogluconate sodium and paromomycin. Additionally, it was observed that oral treatment with cedrol entrapped into NLC displayed higher efficacy in murine visceral leishmaniasis caused by wild or drug-resistant *L.* (*L.) donovani* than cedrol or miltefosine. Thus, the data shown herein indicate that NLC is an interesting platform to deliver drugs, and the formulation UA-NLC was more active than UA in visceral leishmaniasis.

In addition to the antileishmanial activity, UA-NLC also modulated the immune response of golden hamsters with visceral leishmaniasis, increasing the expression of IFN-γ transcripts more efficiently than the groups treated with UA. Effective immunity in leishmaniasis is mediated by IFN-γ, which activates macrophages to a leishmanicidal state [54]. If correctly activated by IFN-γ, macrophages will produce high amounts of the iNOS (inducible nitric oxide synthase) enzyme; that is able to convert nitric oxide (NO) from the precursor L-arginine [55], which has a potent microbicidal potential. In fact, in the present study, it was observed that the increase in IFN-γ mRNA transcript was directly associated with the increase in iNOS expression in animals treated with UA-NLC, suggesting that the expression of both genes along with the leishmanicidal activity of UA cooperated with the marked leishmanicidal activity of this molecule. Of note, in the present work the cytokine quantifications were performed with qPCR, that is an indirect estimate of the bioactive cytokine. Although the amount of RNA can have a direct association with the concentration of the protein, some post-transcriptional mechanisms can regulate the translation of the protein; thus, the real concentration of the bioactive cytokine may be lower than presented in Figure 7.

In addition to the elevation of the cell immune response, it was observed that infected animals treated with 5.0 mg/kg of UA-NLC increased the levels of anti-*Leishmania* IgG, subsequently classified as IgG2 isotype. In experimental murine leishmaniasis, antibodies can be used as markers of resistance, as is the case with IgG2 isotype, which is produced upon the increase in IFN-γ; therefore, it has been associated with resistance and Th1 immune response development [56,57]. In the hamster model, few studies performed this association; however, works of vaccination showed that immunized hamsters develop a Th1 immune response and increase the levels of IgG2 upon challenge with henipavirus [58] or *L. (L.) donovani* [59], suggesting that IgG2 antibody can be a good marker of Th1 immune response as well as resistance in hamsters infected with *L. (L.) infantum.* In contrast, it was observed that animals treated with AmB displayed a significant decrease in IFN-γ and iNOS gene expression, which may be associated with an inhibition of the inflammatory response, caused by the low number of parasites [60]. The treatment with UA-NLC also decreased the number of tissue parasites in the spleen and liver of animals, and contrastingly it stimulated the immune response. Although some works showed that an active infection would maintain an active immune response [60,61], it is still important to observe that UA as well as related pentacyclic triterpene are able to modulate innate and acquired immune responses [62], as observed in treated animals.

In the histopathological study, it was observed that the spleen of the infected control showed expansion of the red pulp, associated with the presence of an elevated number of polymorphonuclear cells; additionally, a higher frequency of macrophage nodules containing intracellular parasites was observed, indicating high disease severity [10]. In contrast, animals treated with UA-NLC showed preservation of white and red pulp, with a low frequency of macrophage nodules, suggesting that this treatment was efficient at decreasing parasitism and controlling the inflammatory response in comparison to the histological sections of animals treated with UA. In the liver of the infected control group, an inflammatory infiltrate was observed in the portal space; however, the intensity of the decreased according to the type of treatment employed, being less intense in the animals treated with UA-NLC when compared with UA. In animals treated with UA-NLC or AmB, a focal inflammatory process was observed, and it was characterized by a discrete infiltration of mononuclear cells in the portal space with low parasitism. The increased leishmanicidal activity and reduced inflammatory process observed in the spleen and liver of animals treated with UA-NLCs in comparison with animals treated with UA can be associated with the uptake of nanoparticles by macrophages, destruction of amastigote forms and the inhibition of inflammation as the number of parasites drastically decreased in the spleen and liver [63]. These data suggest that treatment performed with UA-NLCs preserved the histology of the liver and spleen, since the animals displayed small areas of inflammation in these organs and developed a potent immune response compared to animals treated with UA and nontreated infected groups.

Furthermore, the biochemical parameters related to the assessment of liver and kidney functions reinforce that UA-NLC is safe and can be used in the treatment of visceral leishmaniasis. In fact, it was observed that UA-NLC accentuated the reduction of AST and creatinine in animals with visceral leishmaniasis at levels close to normality. On the other hand, the treatment of infected hamsters with AmB did not normalize the levels of hepatic and renal enzymes, suggesting that treatment, parasite persistence and inflammation are factors associated with liver and kidney damage [64,65]. Although AmB was active in experimental visceral leishmaniasis, the toxicity limits its use. As an alternative, the liposomal version of AmB (AmBisome) should be employed in the present study, allowing for a more robust comparison between the efficacy of UA-NLC and the highly active and safer version of AmB [66,67]. Although, it is still important to note that the costs related to the acquisition of AmBisome are too high, even in the context of preclinical studies. Furthermore, in low-income countries, such as Brazil [68], the treatment of visceral leishmaniasis is performed with conventional AmB; thus, the liposomal version is extremely expensive to be acquired and offered in public medical services.

Taken together, the results obtained herein demonstrate that UA-NLC was stable and showed a homogeneous morphology and size. Additionally, UA-NLC proved to be safe for use in golden hamsters and it showed therapeutic activity in experimental animals with visceral leishmaniasis caused by *L. (L.) infantum*. Importantly, the nanoformulation exhibited superior therapeutic activity than UA given in free form and AmB, which, although it has a significant therapeutic activity, is nephrotoxic, limiting its use in infected animals as well as humans. Thus, the UA loaded into NLCs can be considered an important and promising approach in the treatment of leishmaniasis.

## Figures and Tables

**Table 1:** Particle size (PS), polydispersity (PDI), zeta potential (ZP), and efficacy of encapsulation (EE) of nanostructured lipid carriers (NLC) or UA- NLC. Data are presented as mean ± standard deviation (*n* = 3).

Nanoparticle	PS (nm)	PDI	ZP (mV)	EE (%)
NLC	261.1 ± 3.9	0.16 ± 0.013	−26.12 ± 1.18	
UA-NLC	266.3 ± 5.4	0.18 ± 0.022	−29.26 ± 1.16	59.71 ± 0.2

## Data Availability

Not applicable.
